# A phase II randomized clinical trial using aglycone isoflavones to treat patients with localized prostate cancer in the pre-surgical period prior to radical prostatectomy

**DOI:** 10.18632/oncotarget.27529

**Published:** 2020-04-07

**Authors:** Nagi B. Kumar, Julio Pow-Sang, Philippe Spiess, Shohreh Dickinson, Michael J. Schell

**Affiliations:** ^1^Cancer Epidemiology, H. Lee Moffitt Cancer Center and Research Institute, Inc., Tampa, FL, USA; ^2^Department of Urology, H. Lee Moffitt Cancer Center and Research Institute, Inc., Tampa, FL, USA; ^3^Department of Pathology, H. Lee Moffitt Cancer Center and Research Institute, Inc., Tampa, FL, USA; ^4^Department of Biostatistics, H. Lee Moffitt Cancer Center and Research Institute, Inc., Tampa, FL, USA

**Keywords:** isoflavones, chemoprevention, prostate cancer, African American men, Caucasian men

## Abstract

Prostate cancer (PCa) is the most common cancer in American men. Additionally, African American Men (AAM) are 60% more likely to be diagnosed with PCa and 2.4 times more likely to die from this disease compared to Caucasian men (CM). To date, there are few strategies effective for chemoprevention for men with localized PCa. There is thus a need to continue to evaluate agents and strategies for chemoprevention of prostate cancer. Epidemiological, laboratory and early phase clinical trials have shown that the isoflavones modulates several biomarkers implicated in prostate carcinogenesis. The goal of this phase II randomized clinical trial was to explore the comparative effectiveness and safety of 40 mgs of aglycone isoflavones in AAM and CM with localized PCa in the pre-surgical period prior to radical prostatectomy. Thirty six participants (25 CM, 6AAM) were randomized to the isoflavone arm and 34 (25 CM, 7AAM) to the placebo arm, with 62 completing the intervention. Results indicated that isoflavones at a dose of 20 mgs BID for 3-6 weeks was well tolerated but did not reduce tissue markers of proliferation. A significant reduction in serum PSA was observed with isoflavone supplementation in CM compared to the placebo arm, but not observed in AAM. We observed no changes in serum steroid hormones with isoflavone supplementation. In AAM, a reduction in serum IGF-1 concentrations and IGF1: IGFBP-3 ratios were observed with isoflavone supplementation. Well-powered studies for longer duration of intervention may inform future trials with isoflavones, for chemoprevention of PCa.

## INTRODUCTION

Prostate cancer (PCa) is the most prevalent cancer in American men. The American Cancer Society predicts that in 2019, about 174,650 new cases of PCa will be diagnosed in the United States and that there will about 31,620 deaths associated to this cancer. Approximately 60% of African American Men (AAM) are predicted to be diagnosed with PCa with a mortality rate that is 2.4 times greater compared to Caucasian Men (CM) [1, 2]. It is estimated that 29,570 AAM will be diagnosed with prostate cancer in 2019 and 5,350 will die of the disease. PCa has a long latency period, characterized by abnormal differentiation of cells and tissue [2] and propelled by genetic and epigenetic changes [2–4]. Although reports have demonstrated a significant variation in PCa morbidity and mortality rates in population studies, the incidence of pre neoplastic lesions [5, 6] and PCa in autopsies [7] are comparable, implying the influence of other environmental factors in the etiology of this disease and the potential to prevent PCa utilizing pharmacological approaches [8–10] Large phase III trials evaluating strategies for PCa chemoprevention have included 5-alpha-reductase inhibitors, finasteride and dutasteride [11–13]. However, results indicated an increased detection of high-grade PCa, limiting their clinical adoption [12]. Other agents for PCa chemoprevention evaluated included the trace mineral selenium and Vitamin E, either as single agents or in combination. Both selenium and Vitamin E showed no chemoprevention benefit. On the other hand, a significant increase in PCa was observed in the treatment arm receiving vitamin E alone [14]. Currently, strategies using dietary interventions (The Men’s Eating and Living (MEAL) Study [15] as well as a trial evaluating pomegranate fruit extract [16] targeting men with localized PCa have not appeared promising, Thus, to date, although several agents have been evaluated for safety and efficacy in laboratory and early phase trials [17–19], there is minimal evidence available of any one agent or strategy that has been found to be effective for primary and secondary chemoprevention of PCa. Our approach for chemoprevention for PCa is to use a rigorous and broad spectrum approach [20] by utilizing agents that have (a) bioavailability; (b) safety; (c) target relevant and multiple molecular pathways in a robust manner; and (d) modulate intermediate endpoint biomarkers implicated progression of PCa. We propose that this broad spectrum approach may be more efficacious that those approaches and agents evaluated to date.

Epidemiological and laboratory studies have demonstrated that multiple phytochemicals, including isoflavones, could induce apoptosis, controlling the formation and growth of human cancers, including PCa [20–25] with an acceptable safety profile, making them appealing candidates for chemoprevention of PCa. A link between isoflavones and PCa was first observed in populations consuming a significant amount of dietary soy products which was associated with a lower risk of prostate cancer [6, 7]. Additionally, studies in Asian men have reported an inverse relationship between dietary intake of soy, plasma isoflavones [20–23, 25] and prostatic fluid [24] concentrations of isoflavones and the incidence of PCa. On the other hand, migration studies of Asian men have reported increased risk of PCa, epically among those men who abandon their original dietary habits upon migrating to the U.S. [26–28]. Similarly, PCa incidence has increased in Asian countries concurrently with westernization of their diets [29].

Among the isoflavones found in soy and other foods, genisteins has been observed to be the most abundant, biologically active and a potent modulator of prostate carcinogenesis compared to other [2] isoflavones, daidzein and glycitein [4]. Isoflavones including genistein, daidzein and glycitein have been identified in foods such as soy, other legumes and plants, such as red clover [2, 4]. *In vitro* data have consistently shown that genistein demonstrates both genomic and non-genomic effects, modulating cell proliferation [30–34], angiogenesis [35, 36], tumor cell invasion and tumor metastasis [32, 37, 38] cell cycle regulation [38], antioxidant [37, 39] induces apoptotic cell death [40], functions critical for chemoprevention. Other functions of genistein include the anti-inflammatory properties by decreasing COX-2 mRNA and protein levels in cancer cells, reduction in the secretion of prostaglandin E2 (PGE2) and reduced mRNA levels of the prostaglandin receptors EP4 and FP, suggesting that genistein may exert chemopreventive effects by inhibiting the synthesis of prostaglandins, which promote inflammation [41]. Genistein and daidzein treated PCa cells have been shown to downregulate growth factors involved in angiogenesis (e. g., EGF and IGF-1) and the interleukin-8 gene, associated with cancer progression [42]. We have previously reported that isoflavone, genistein, induce apoptosis and inhibit growth in both androgen-sensitive and androgen independent PCa cells [40].

Early phase I trials in healthy, early-stage or treated cancer patient cohorts have demonstrated the, pharmacokinetics safety and clinical characteristics of whole soy and isoflavones, administered as whole soy products or individual isoflavones [43–46]. A few pilot studies, including our earlier studies [35–37], have shown some reductions in steroid hormones and stabilization or reduction of prostate specific antigen (PSA) by isoflavones [45, 47–52]. With African American men being at highest risk of PCa, in addition to examining the safety and effectiveness of specific effects of 40 mgs of aglycone isoflavones on intermediate endpoint biomarkers implicated in PCa progression, our goal was to explore the comparative effectiveness and safety of isoflavones for PCa chemoprevention in AAM and CM. We report below the methods, results and discussion of a phase II randomized, double-blind, placebo controlled trial that examined the comparative safety and effectiveness of a standardized formulation of 40 mgs of aglycone isoflavones per day, in AAM and CM with localized PCa in the pre-surgical period preceding a scheduled radical prostatectomy.

## RESULTS

**Figure 1:**
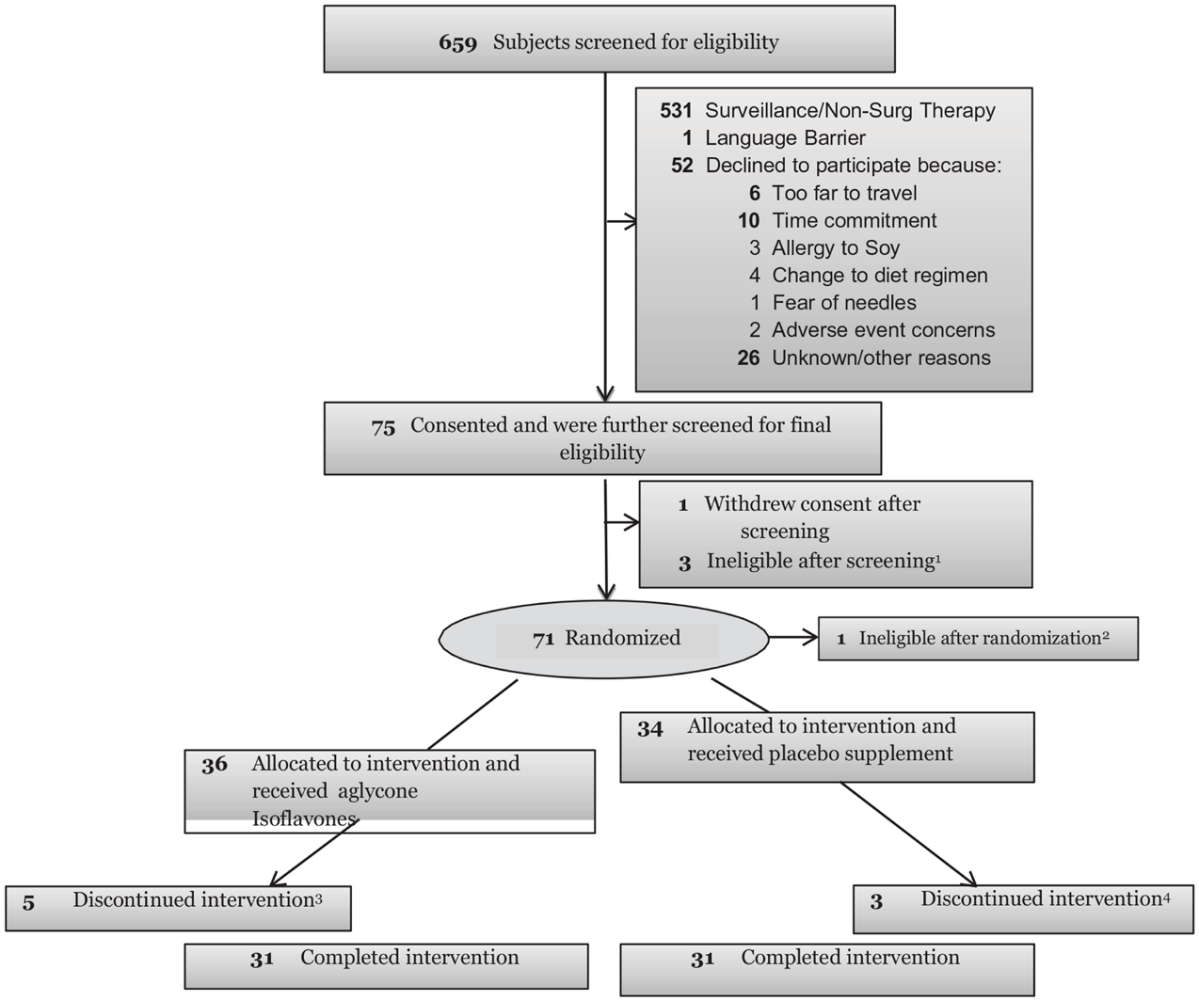
Consort diagram. ^1^Previously undetected exclusionary medical factors included 3 patients with elevated kidney function results. ^2^Pathology review showed no presence of CaP. ^3^3subjects withdrew consent and 2 off study due to AE (1 unlikely related and 1 probably related to study). ^4^2 subjects withdrew consent; 1 off study due to AE (possibly related to study).

**Figure 2:**
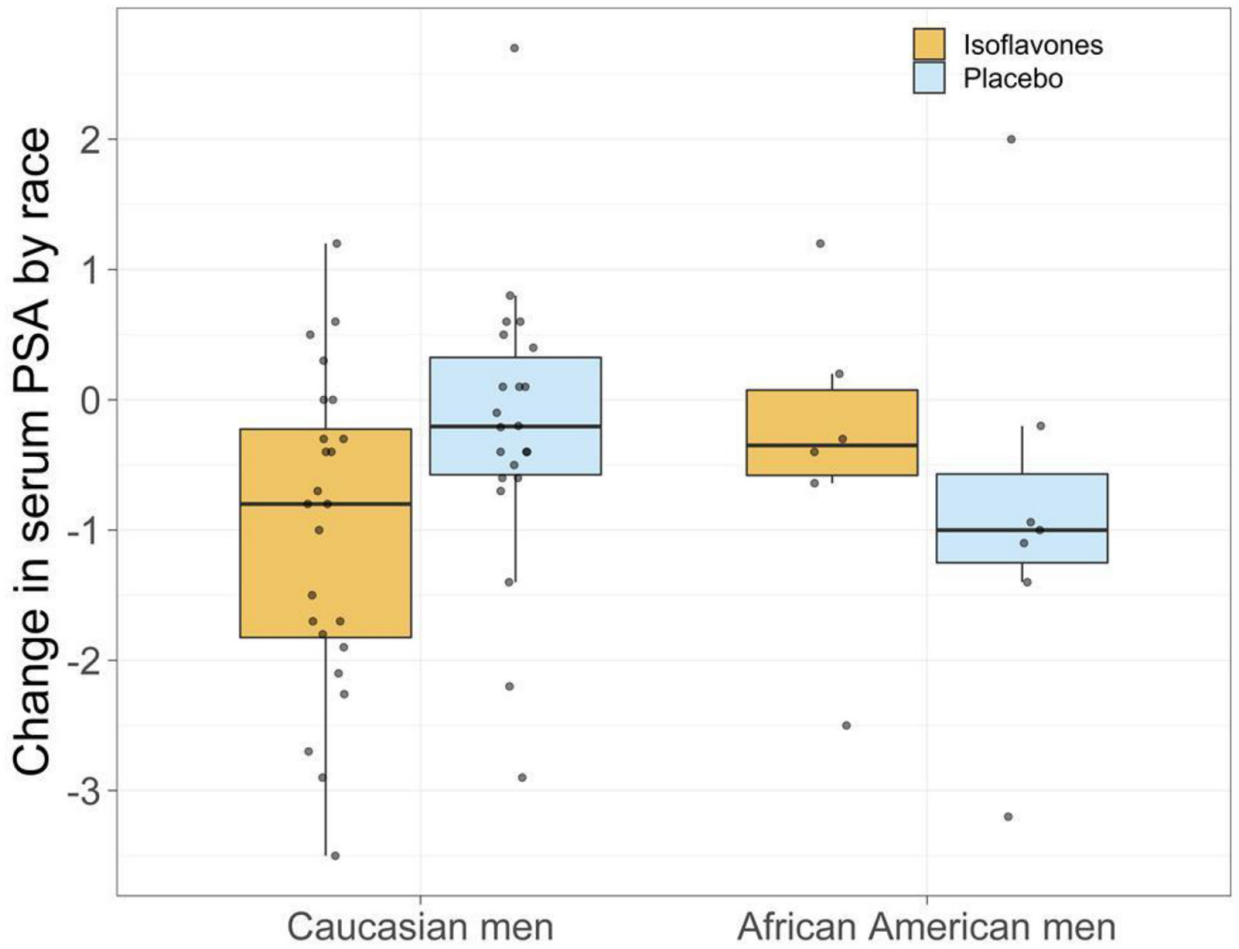
Change in serum PSA by race.

**Figure 3:**
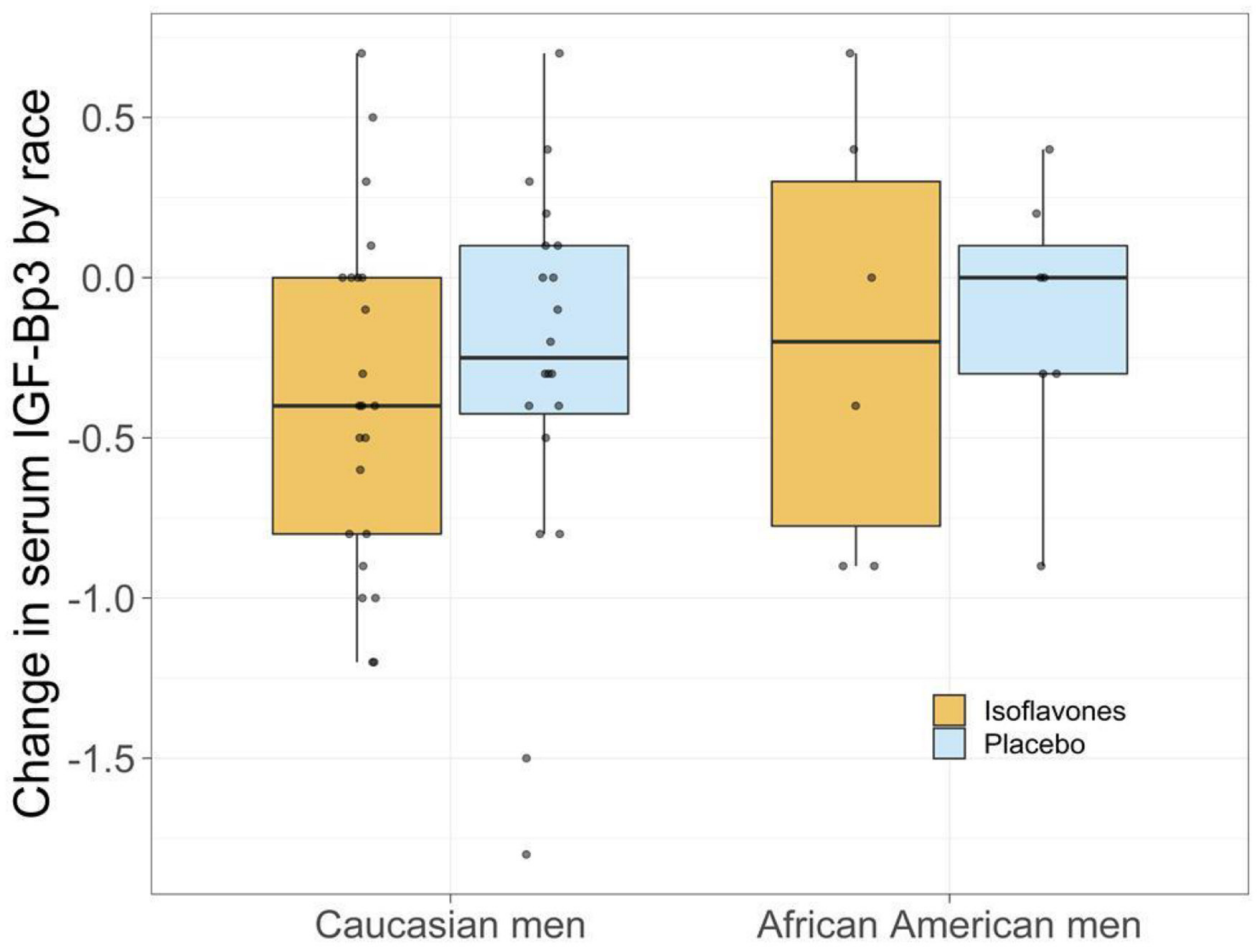
Change in serum IGF1-IGF Bp3 by race.

Of a total of 128 men meeting all eligibility requirements, 71 were randomized on study (Figure 1). Thirty six participants (25 CM, 6AAM) were randomized to the isoflavone arm and 34 (25 CM, 7AAM) to the placebo arm, with 62 completing the intervention (Figure 1). Although we experienced significant challenges to recruitment, we were able to retain 87% of the subject recruited to the study. Table 1 displays the baseline characteristics of all study participants. The 2 study arms were well matched for potential predictive markers, including age, race, ethnicity, PSA and body mass index (BMI). Criteria for inclusion included only men with Gleason 6. Although pill count and compliance were monitored to ensure compliance to agent, the plasma concentrations of specific isoflavones were not reflective of isoflavone intake, including genistein (Table 2). Other isoflavones were non-detectable or below quantifiable levels in the plasma of all subjects and thus not reported. Additionally, no significant change in intake of specific nutrients from baseline to the end of study was observed, indicating that compliance was maintained on both study arms (data not shown). A summary of all toxicities by final attribution appears in Table 3A and 3B. Overall, isoflavones at a dose of 40 mgs/day administered in divided doses of 20 mgs of aglycones with meals was well tolerated. All adverse events were limited to grade I-II events in the two study arms with the exception of one (1) grade III event in the placebo arm considered not related to study agent. Adverse events did not vary by race. All grade I-II gastrointestinal symptoms were considered possibly or probably related to study agent, although subjects recovered and continued on study agent. Changes from baseline to post-intervention of serum PSA is displayed in Table 4A–4C. However, with the current duration of intervention and sample size, we failed to observe statistically significant reduction is serum PSA. However, among CM, greater reduction in serum PSA was observed in the treatment arm compared to the placebo arm (*P* = 0.03) (Figure 2). Mean changes in serum steroid hormone concentrations of estradiol, total and free testosterone, sex-hormone binding globulin, IGF-1 and IGFBP-3 from baseline to post intervention by treatment arms and race is presented in Table 5A–5C. Overall, we observed no changes in steroid hormone levels and SHBG in the current trial. Although we observed a trend towards a decrease in free testosterone and increase in SHBG in the treatment arm compared to the placebo arm, this difference was not statistically significant for this duration of intervention and sample size. We did not observe any differences between treatment and placebo arms for serum concentrations of estradiol, total and free testosterone in the AAM and CM subgroups. However, IGF-1: IGF Binding Protein -3 Ratio decreased in the isoflavone treated arm in AAM and increased in the placebo arm. (Figure 3). Although the baseline and post treatment prostate cancer tissue was unavailable from all the subjects in the study and the sample size was small, a trend was observed demonstrating that median increase in Ki-67 expression was much lower in the isoflavone treated arm compared to the placebo arm of the study. (Table 6A) Although we analyzed these changes by race, due to the unavailability of tissue from all subjects in the study, no conclusions could be reached (Table 6B and 6C). We did not observe significant changes from baseline to post intervention in both arms of the study in Gleason Score or Tumor volume (data not shown). No significant differences were observed in LUTS scores from baseline to end of study between the two study arms. (Data not shown).

**Table 1:** Demographic characteristics of all study participants randomized to the clinical trial (*N* = 71)

Variables	Levels	Isoflavones (*N* = 36)	Placebo (*N* = 35)	*P* value^*^
		*N* (%)	*N* (%)	
Age (years)	Mean (SD)	58.8 (7.5)	59.1 (7.4)	0.73
Race	Black Or African American	7 (20.0)	8 (24.2)	0.77
	White	28 (80.0)	25 (75.8)	
Ethnicity	Hispanic	5 (13.9)	2 (5.7)	0.57
	Non-Hispanic	30 (83.3)	31 (88.6)	
	Unknown	1 (2.8)	2 (5.7)	
Family History of Prostate Cancer	N			
	Y			
Body Mass Index (Weight in Kgs/height in m^2^)	Mean (SD)	30.1 (4.0)	31.2 (5.0)	0.49
PSA (ng/ml)	Mean (SD)	6.4 (2.9)	5.8 (3.2)	0.26

Abbreviations: PCa: Prostate Cancer, PSA, prostate-specific antigen; SD, standard deviation. ^*^ *P* values were computed by Fisher’s exact test for categorical variables, Wilcoxon rank-sum test for continuous variables.

**Table 2:** Plasma concentrations of isoflavone-Genistein from baseline to post-intervention by study arm

Treatment	Time (*N*)	Genistein (mg) Median (Min/Max)	*P* value of change from Baseline^*^
**Placebo**	Baseline (*N* = 28)	0.15 (0.00/2.89)	0.40
	End of study (*N* = 28)	0.06 (0.00/1.53)	
	Change from baseline (*N* = 27)	–0.02 (–1.36/0.47)	
**Isoflavones**	Baseline (*N* = 29)	0.07 (0.00/3.08)	
	End of study (*N* = 31)	0.07 (0.00/0.47)	
	Change from baseline (*N* = 29)	0.00 (–3.06/0.37)	

^*^Wilcoxon rank sum test.

**Table 3A:** Number of toxicities events by final attribution and treatment arm - all patients (*N* = 71)

Table of Attribution by Treatment			
Attribution	Treatment		
	Placebo N (row%)	Soy Isoflavones N (row%)	Total
**Possible**	37	37	74
	50.00	50.00	
**Probable**	0	4	4
	0.00	100.00	
**Unlikely**	9	17	26
	34.62	65.38	
**Unrelated**	20	24	44
	45.45	54.55	
**Total**	66	82	148

**Table 3B:** Summary of toxicity by maximum grade - related toxicities (attribution = definite, probable, possible, not related)

Drug Administration	Toxicity Category	Description (CDUS Toxicity Type Code)	Grade 1 *n* (%)	Grade 2 *n* (%)	Grade 3 *n* (%)	Grade 4 *n* (%)	Grade 5 *n* (%)	All *n* (%)	N^*^
Placebo	Abdominal pain	Stomach cramps	—	1 (2.9)	—	—	—	1 (2.9)	35
	Aspartate aminotransferase increased	Elevated AST	1 (2.9)	—	—	—	—	1 (2.9)	35
	Atrial fibrillation	Atrial Fibrillation	—	1 (2.9)	—	—	—	1 (2.9)	35
	Bloating	Bloating	—	1 (2.9)	—	—	—	1 (2.9)	35
	Blood bilirubin increased	Elevated Direct Bilirubin	1 (2.9)	—	—	—	—	1 (2.9)	35
	Constipation	Constipation	1 (2.9)	—	—	—	—	1 (2.9)	35
	Diarrhea	Diarrhea	—	1 (2.9)	—	—	—	1 (2.9)	35
		Occasional Diarrhea	1 (2.9)	—	—	—	—	1 (2.9)	35
	Gastrointestinal disorders - Other, specify	Belching	1 (2.9)	—	—	—	—	1 (2.9)	35
		GI Symptoms	1 (2.9)	—	—	—	—	1 (2.9)	35
	Hyperglycemia	Elevated Glucose	1 (2.9)	—	—	—	—	1 (2.9)	35
		Hyperglycemia	2 (5.7)	1 (2.9)	—	—	—	3 (8.6)	35
	Hypocalcemia	Hypocalcemia	1 (2.9)	—	—	—	—	1 (2.9)	35
	Hyponatremia	Hyponatremia	1 (2.9)	—	—	—	—	1 (2.9)	35
	Intraoperative hemorrhage	Hemorrhage / Bleeding	—	—	1 (2.9)	—	—	1 (2.9)	35
	Investigations - Other, specify	Elevated Total Testosterone	1 (2.9)	—	—	—	—	1 (2.9)	35
		Low Hemoglobin	1 (2.9)	—	—	—	—	1 (2.9)	35
		Low RBC	2 (5.7)	—	—	—	—	2 (5.7)	35
	Metabolism and nutrition disorders - Other, specify	Low Carbon Dioxide	3 (8.6)	—	—	—	—	3 (8.6)	35
		Low Carbon dioxide	1 (2.9)	—	—	—	—	1 (2.9)	35
		Low Globulin	1 (2.9)	—	—	—	—	1 (2.9)	35
		Low Protein	1 (2.9)	—	—	—	—	1 (2.9)	35
	Nausea	Nausea	1 (2.9)	—	—	—	—	1 (2.9)	35
	Neutrophil count decreased	Low Absolute Neutrophils	1 (2.9)	—	—	—	—	1 (2.9)	35
	Pain in extremity	Arthritic Pain in Finger	—	1 (2.9)	—	—	—	1 (2.9)	35
	Renal and urinary disorders - Other, specify	Urination Discomfort	1 (2.9)	—	—	—	—	1 (2.9)	35
	Sinusitis	Sinus infection	—	1 (2.9)	—	—	—	1 (2.9)	35
	Sore throat	Sore throat	—	1 (2.9)	—	—	—	1 (2.9)	35
	Stomach pain	Stomach cramps	—	1 (2.9)	—	—	—	1 (2.9)	35
		Tender stomach	—	1 (2.9)	—	—	—	1 (2.9)	35
	Urticarial	Hives / Rash	1 (2.9)	—	—	—	—	1 (2.9)	35
Soy isoflavones	Aspartate aminotransferase increased	Elevated AST	2 (5.6)	—	—	—	—	2 (5.6)	36
	Blood bilirubin increased	Elevated direct bilirubin	1 (2.8)	—	—	—	—	1 (2.8)	36
		Elevated total bilirubin	1 (2.8)	—	—	—	—	1 (2.8)	36
	Constipation	Constipation	1 (2.8)	—	—	—	—	1 (2.8)	36
	Diarrhea	Diarrhea	—	2 (5.6)	—	—	—	2 (5.6)	36
	Fatigue	Fatigue	1 (2.8)	—	—	—	—	1 (2.8)	36
	Flatulence	Gas	3 (8.3)	—	—	—	—	3 (8.3)	36
	Gastrointestinal disorders - Other, specify	Darkening of stool	1 (2.8)	—	—	—	—	1 (2.8)	36
		Soft stool	1 (2.8)	—	—	—	—	1 (2.8)	36
	General disorders and administration site conditions - Other, specify	Feeling of fullness	1 (2.8)	—	—	—	—	1 (2.8)	36
	Hyperglycemia	Elevated Glucose	1 (2.8)	—	—	—	—	1 (2.8)	36
	Hypernatremia	Elevated Sodium	1 (2.8)	—	—	—	—	1 (2.8)	36
	Hypocalcemia	Low Calcium	1 (2.8)	—	—	—	—	1 (2.8)	36
	Investigations - Other, specify	Elevated Estradiol	1 (2.8)	—	—	—	—	1 (2.8)	36
		Low Carbon Dioxide	1 (2.8)	—	—	—	—	1 (2.8)	36
		Low IGF Binding Protein 3	1 (2.8)	—	—	—	—	1 (2.8)	36
		Low Serum Co2	1 (2.8)	—	—	—	—	1 (2.8)	36
		Low total testosterone	1 (2.8)	—	—	—	—	1 (2.8)	36
	Metabolism and nutrition disorders - Other, specify	Elevated AST	1 (2.8)	—	—	—	—	1 (2.8)	36
		Elevated Albumin	1 (2.8)	—	—	—	—	1 (2.8)	36
		Low Carbon Dioxide	4 (11.1)	—	—	—	—	4 (11.1)	36
		Low Creatinine	1 (2.8)	—	—	—	—	1 (2.8)	36
		Low creatinine	1 (2.8)	—	—	—	—	1 (2.8)	36
	Nausea	Nausea	—	1 (2.8)	—	—	—	1 (2.8)	36
	Pain in extremity	Cramps in hand	1 (2.8)	—	—	—	—	1 (2.8)	36
	Renal and urinary disorders - Other, specify	Slow stream	1 (2.8)	—	—	—	—	1 (2.8)	36
	Stomach pain	Stomach ache	1 (2.8)	—	—	—	—	1 (2.8)	36
	Urinary frequency	Frequent urination	1 (2.8)	—	—	—	—	1 (2.8)	36
		Frequent urination (night)	1 (2.8)	—	—	—	—	1 (2.8)	36
	White blood cell decreased	Low WBC	1 (2.8)	—	—	—	—	1 (2.8)	36
All	Overall	All	23 (32.4)	7 (9.9)	1 (1.4)	—	—	31 (43.7)	71

^*^N: Number of subjects treated and evaluable for toxicity.

**Table 4A:** Change in serum PSA (ng/mL) from baseline to post-intervention by study arm

Treatment (N)	Time (weeks)	Median (Min/Max)	*P* value of change from Baseline^*^
**Placebo**	Baseline (*N* = 35)	5.5 (0.6/13.6)	0.072
	End of study (*N* = 31)	5.2 (0.6/15.6)	
	Change from baseline (*N* = 31)	–0.2 (–3.2/2.7)	
**Isoflavones**	Baseline (*N* = 35)	6.2 (2.1/17.8)	
	End of study (*N* = 31)	4.9 (2.0/14.3)	
	Change from baseline (*N* = 31)	–0.7 (–3.5/1.2)	

**Table 4B:** Change in serum PSA (ng/mL) from baseline to post-intervention by race (CM only)

Treatment (N)	Time (weeks)	Median (Min/Max)	*P* value of change from Baseline^*^
**Placebo**	Baseline (*N* = 25)	4.8 (0.6/10.1)	
	End of study (*N* = 22)	5.2 (0.6/10.2)	
	Change from baseline (*N* = 22)	–0.2 (–2.9/2.7)	0.03
**Isoflavones**	Baseline (*N* = 27)	6.4 (2.1/17.8)	
	End of study (*N* = 24)	5.4 (2.1/14.3)	
	Change from baseline (*N* = 24)	–0.8 (–3.5/1.2)	

**Table 4C:** Change in serum PSA (ng/mL) from baseline to post-intervention by race (AAM only)

Treatment	Time (weeks) (N)	Median (Min/Max)	*p* value of change from Baseline^*^
**Placebo**	End of study (*N* = 7)	8.2 (3.2/15.6)	
	Change from baseline (*N* = 7)	–1.0 (–3.2/2.0)	
**Isoflavones**	Baseline (*N* = 7)	4.3 (2.4/8.0)	0.35
	End of study (*N* = 6)	4.1 (2.0/5.6)	
	Change from baseline (*N* = 6)	–0.3 (–2.5/ 1.2)	

^*^Wilcoxon rank-sum test, CM: Caucasian men, AAM: African American men.

**Table 5A:** Mean change in steroid hormones from baseline to post-intervention by study arm (*N* = 71 for the variable with least missing data)

Variable	Isoflavones Pre-Tmt	Isoflavones Post-Tmt	*p* value^*^	Placebo Pre-Tmt	Placebo Post-Tmt	*p* value^*^	Isoflavones arms vs. Placebo p^**^
	Median (Min/Max)	Median (Min/Max)		Median (Min/Max)	Median (Min/Max)		
Estradiol pmo/L	37.5 (18.0/67.0)	39.0 (15.0/71.0)	0.66	37.0 (17.0/67.0)	39.5 (17.0/ 60.0)	0.57	0.96
Free testosterone (pg/ml)	59.9 (21.3/101)	54.8 (17.6/119)	0.61	49.1 (30.4/134)	62.9 (24.8/ 106)	0.084	0.45
IGF Binding Protein –3 (mg/L)	3.9 (1.8/5.5)	3.8 (1.7/4.7)	0.0043	4.3 (1.7/ 6.4)	4.2 (2.0/ 5.6)	0.066	0.33
IGF-1 (ng/mL)	160 (0.0/288)	153 (53.0/258)	0.22	143 (52.0/285)	143 (71.0/ 301)	0.32	0.12
SHBG nmol/L	34.0 (14.0/92.0)	35.0 (13.0/61.0)	0.63	32.0 (12.0/ 110)	32.0 (14.0/ 81.0)	0.37	0.32
Total Testosterone (ng/dL)	358 (93.0/738)	376 (163/709)	0.88	348 (179/ 979)	295 (196/1157)	0.52	0.56
IGF-1 (ng/mL): IGF Binding Protein -3 (mg/L): Ratio	36.6 (22.0/62.4)	40.2 (20.4/74.0)	0.53	40.5 (0.0/ 62.6)	41.9 (20.4/61.2)	0.012	0.13

^*^Wilcoxon signed rank test, ^**^Wilcoxon rank-sum test, Tmt: treatment.

**Table 5B:** Mean change in steroid hormones from baseline to post-intervention by race (CM only) (*N* = 53 for the variable with least missing data)

Variable	Isoflavones Pre-Tmt	Isoflavones Post-Tmt	*p* value^*^	Placebo Pre-Tmt	Placebo Post-Tmt	*p* value^*^	Isoflavones arms vs. Placebo p^**^
	Median (Min/Max)	Median (Min/Max)		Median (Min/Max)	Median (Min/Max)		
Estradiol pmo/L	37.5 (18.0/63.0)	40.0 (15.0/71.0)	0.60	37.0 (18.0/57.0)	41.0 (21.0/60.0)	0.27	0.7442
Free testosterone (pg/ml)	59.9 (21.3/101)	51.8 (17.6/119)	0.65	47.7 (30.4/125)	61.6 (24.8/91.2)	0.24	0.8510
IGF Binding Protein -3 (mg/L)	4.1 (1.8/5.5)	3.6 (1.7/4.7)	0.0036	4.4 (2.1/6.4)	4.3 (2.0/5.6)	0.056	0.3350
IGF-1 (ng/mL)	167 (0.0/288)	153 (78.0/258)	0.47	160 (97.0/285)	157 (71.0/301)	0.79	0.4731
SHBG nmol/L	37.0 (15.0/92.0)	43.5 (13.0/61.0)	0.60	32.0 (12.0/79.0)	32.0 (17.0/51.0)	0.76	0.5921
Total Testosterone (ng/dL)	377 (93.0/738)	411 (167/709)	0.67	346 (179/705)	295 (196/608)	0.81	0.8916
IGF-1 (ng/mL): IGF Binding Protein -3 (mg/L): Ratio	40.5/ (0.0/62.6)	41.9 (30.0/61.2)	<.0001	37.1 (22.0/62.4)	42.8 (20.4/74.0)	<.0001	0.7642

**Table 5C:** Mean change in steroid hormones from baseline to post-intervention by race (AAM only) (*N* = 15 for variables with least missing values)

Variable	Isoflavones Pre-Tmt	Isoflavones Post-Tmt	*p* value^*^	Placebo Pre-Tmt	Placebo Post-Tmt	*p* value^*^	Isoflavones arms vs. Placebo p^**^
	Median (Min/Max)	Median (Min/Max)		Median (Min/Max)	Median (Min/Max)		
Estradiol pmo/L	38.0 (26.0/67.0)	36.0 (15.0/61.0)	0.66	28.5 (17.0/67.0)	27.0 (17.0/46.0)	0.31	1.0000
Free testosterone (pg/ml)	57.2 (31.1/92.6)	58.7 (34.6/103)	0.69	57.4 (41.8/134)	79.5 (35.9/106)	0.38	0.4555
IGF Binding Protein -3 (mg/L)	3.9 (3.0/4.8)	3.9 (2.6/4.6)	0.56	3.3 (1.7/5.4)	3.4 (2.3/4.5)	0.63	1.0000
IGF-1 (ng/mL)	147 (96.0/287)	104 (53.0/229)	0.44	107 (52.0/219)	119 (95.0/211)	0.56	0.1038
SHBG nmol/L	25.0 (17.0/41.0)	24.5 (17.0/33.0)	0.75	46.0 (14.0/110)	31.0 (14.0/81.0)	0.56	0.5168
Total Testosterone (ng/dL)	304 (202/712)	332 (163/493)	0.22	464 (215/979)	381 (214/1157)	1	0.5228
IGF-1 (ng/mL): IGF Binding Protein -3 (mg/L): Ratio	37.7 (32.0/59.8)	27.4 (20.4/50.0)	0.19	35.1 (24.7/49.7)	44.1 (24.9/51.7)	0.11	0.0740

^*^Wilcoxon signed rank test. ^**^Wilcoxon rank-sum test.

**Table 6A:** Change in median Ki-67 (primary outcome variable) from baseline to post-intervention by study arm

Treatment (*N*)	Time (weeks)	Median (Min/Max)	*p* value^*^
**Placebo**	Baseline (*N* = 14)	1.0 (0.0/2.0)	0.072
	End of study (*N* = 13)	2.5 (2.0/6.0)	
	Change from baseline (*N* = 11)	1.0 (0.0/6.0)	
**Isoflavones**	Baseline (*N* = 12)	1.5 (0.0/6.0)	
	End of study (*N* = 16)	2.0 (1.0/6.0)	
	Change from baseline (*N* = 11)	0.5 (0.0/2.0)	

^*^Wilcoxon rank-sum test.

**Table 6B:** Change in median Ki-67 (primary outcome variable) from baseline to post-intervention by race (CM only)

Variable	ARM of study	*N*	Median (Min/Max)	*p* value^*^
Median% Ki67 change	Placebo	9	1.0 (0.0/6.0)	0.1086
	Soy isoflavone	9	1.0 (0.0/2.0)	

^*^Wilcoxon rank-sum test.

**Table 6C:** Change in median Ki-67 (primary outcome variable) from baseline to post-intervention by race (AAM only)

Variable	ARM of study	*N*	Median (Min/Max)	*p* value^*^
Median % Ki67 change	Placebo	1	1.0 (1.0/1.0)	0.5403
	Soy isoflavone	2	0.3 (0.0/0.5)	

^*^Wilcoxon rank-sum test.

## DISCUSSION

Our study, to our knowledge, was the first to evaluate comparative effectiveness and safety of isoflavones in between AAM and CM in a randomized, placebo-controlled clinical trial of isoflavones in men with localized PCa. In this Phase II clinical trial, we were able to demonstrate that in an AA and Caucasian cohort of men with localized PCa, we were able to achieve compliance to a daily regimen of study agent/placebo and diet. Subjects in the study were able to maintain a weekly diet, and symptom records. Subjects attended follow up visits, study related interviews and were able to obtain blood draws that were planned to monitor compliance to study agent and to monitor, safety and toxicity. However, other than significant decreases in serum PSA observed in CM compared to AAM, there were no other differences in other neither biological nor clinical biomarkers observed between the two (2) races.

Several epidemiological studies have demonstrated a high correlation between increasing dose of isoflavone intake with plasma, serum and urinary metabolites of isoflavones [43, 53, 54]. Other studies have reported significant variations in isoflavone absorption, attributing these variations to intake of other foods that potentially have an impact on gut bacteria and seasonal changes in nutrient composition of foods. Additionally, the timing of plasma and urinary isoflavone measurements may produce variability due to the short half-life of these compounds, the composition of ethnic diets, and individual differences [43–45, 53–57]. In a report to estimate bioavailability of usual intake of isoflavones, Bakta et al. (2004) [53] report stable plasma isoflavone levels over a one year period in populations who consume a steady intake of food sources of isoflavones with no significant increases over time. Similarly, animal models using serial measurements of plasma isoflavones have failed to observe significant and progressive increase in plasma concentrations with a chronic dose of purified isoflavones [58]. Other studies have demonstrated that urinary excretion of isoflavone metabolites increases with dose, frequency and length of time the supplement is used, demonstrating a higher elimination of metabolites of isoflavones with increasing length of use [35, 56, 58], indicative of a trend towards stabilization of bioavailability concentration of isoflavones with time. Adherence to agent/placebo was greater than 90% as indicated by pill count, self-reported agent logs and plasma isoflavone levels, although the increase in plasma isoflavones failed to reflect intake in the treatment arm. Our findings establish the need for further investigations into the pharmacokinetics of isoflavones in long-term clinical trials that includes evaluation of the influence of gut microbiota in isoflavone absorption. Other than gastrointestinal symptoms that were reported by both study arms, all other grade I-II toxicities were determined from monthly CMP panels.

Change in percent %Ki-67 expression evaluated in PCa tissue specimens at baseline to end of intervention (+/-3 days) with isoflavones (40 mg aglycone isoflavones daily) *vs*. placebo. Ki67, a nuclear protein found in all phases of the cell cycle can provide valuable information about the nature and progression of not only aggressive prostate cancers but also the indolent variants. Ki67 expression is magnified in prostatic carcinoma and remains low in benign and normal prostatic cells [19, 59], identifying it as an important and informative biomarker for calibrating the biological effects of isoflavones in modulating disease progression. Although change in % Ki-67 expression could potentially be applied as an intermediate endpoint biomarker of chemoprevention efficacy, it has not been previously monitored serially (from start to end of chemoprevention intervention) in chemoprevention trials comparing effectiveness of isoflavones between AAM and CM [50, 60]. Overall, we did not observe a robust decrease in Ki-67 in the treatment arm compared to placebo. However, we observed a trend demonstrating that median increase in Ki-67 expression was much lower in the isoflavone treated arm compared to the placebo arm of the study.

Although steroid hormone have been implicated in the etiology of prostate cancer, the mechanism by continues to be unclear. Patients with PCa have been observed to have higher free testosterone (unbound) levels and lower levels of sex-hormone binding globulin (SHBG), estrone [21] and estradiol [61]. Androgens are essential for the function and growth of the prostate and are known to stimulate the proliferation of human prostatic cells [61–64]. Administration of hormonal therapies has been shown to produce PCa in rodents, while castration, anti-androgens and leuteinizing hormone-releasing hormone (LH-RH) agonists’ therapy can reduce PCa progression [62–64]. It is clear from recent studies that testosterone and estradiol are important contributors of androgenic and estrogenic activity [62–64]. The role of estrogens in the treatment of androgen-independent PCa’s have recently been examined in Phase II clinical trials, demonstrating that high dose Premarin^®^ resulted in prostate specific antigen decreases in patients with androgen independent PCa [65, 66]. In a previous clinical trial, we observed a significant increase in serum total estradiol in the 40 mgs isoflavone-treated arm (*P* = 0.02) and not at higher doses (60, 80 mgs) of isoflavones [50]. However, no change in serum estradiol with isoflavones was observed in the current study. Although we observed a trend towards a decrease in free testosterone in the treatment arm compared to the placebo arm, this difference was not statistically significant for this duration of intervention and sample size. No differences between treatment and placebo arms were observed for serum estradiol, total and free testosterone in the AAM and CM subgroups. Our results are consistent with previous randomized clinical trials including a meta-analysis of 32 studies (12) where changes in serum steroid hormones have not been observed.

Sex-hormone binding globulin has been shown to have a protective effect in prostate cancer, by potentially impacting the estrogen/testosterone balance by binding to and sequestering testosterone and estradiol. This mechanism of action has, in addition shown to control the bioavailability of these steroid hormones to target cells [62]. Others have shown that SHBG can function as a hormone with a direct interaction with prostate cells [64]. Overall, we failed to observe statistically significant modulation of these steroid hormones and SHBG in this trial for this duration of intervention and sample size.

The role of the insulin-like growth factor (IGF) axis and interaction with androgen-suppressing agents in relation to prostate carcinogenesis continues to be unclear. The insulin-like growth factor 1 (IGF-1) and members of the IGF-binding protein family (IGFBPs) are essential for cell cycle regulation, with mitogenic and anti-apoptotic functions. However, these functions are tempered by binding to IGFBPs [67, 68], as much of the circulating IGF-1 is bound to IGFBPs (mainly IGFBP-3). A very small percentage of IGF-1 remains in an unbound and biologically active form. 2 Multiple factors such as gender, race, nutrition, lifestyle and age affect IGF-1 and IGFBP-3 levels which are primarily controlled by pituitary growth hormone (GH), and affected by multiple factors. The bioavailability of IGF1 is indicated by the IGF1/IGFBP3 molar ratio. The IGF-1 receptor (IGF-IR) has been implicated in PCa progression and progression to androgen-independent (AI) disease, as an androgens have been demonstrated to up-regulate insulin-like growth factor-I receptor (IGF-IR) expression and sensitize PCa cells to the biological effects of IGF-I. IGF-1 levels increase and IGFBP3 protein levels decrease during the progression of PCa [67–70]. We observed a reduction in serum IGF-1 in the isoflavone-treated group compared to placebo, although these reductions were not statistically significant. The median values in both treatment arms were nearly the same. A significant increase in IGFBP-3 was observed in both study arms in this trial potentially indicative of a study population with low grade disease since higher serum IGFBP3 is observed in low-grade disease [71]. However, IGF-1: IGF Binding Protein -3 Ratio decreased in the isoflavone treated arm in AAM and increased in the placebo arm. Since IGF1/IGFBP3 ratio is an indicator of IGF1 bioavailability, an observation of a trend towards reduction in the IGF1/IGFBP3 ratio and serum IGF-1 observed in AAM men treated with isoflavones compared to placebo is interesting and may indicate decreased bioavailability of IGF-1 with treatment. These early observations must be verified in future well-powered studies.

The value of PSA changes in a chemoprevention setting is debatable. Despite this drawback, serum PSA as a continuous variable has been widely used in PCa chemoprevention trials [72–74] as well as in clinical practice, where PSA levels are used to define risk categories [75–78]. However, with the current duration of intervention and sample size, we failed to observe statistically significant reduction is serum PSA. However, among CM, greater reduction in serum PSA was observed in the treatment arm compared to the placebo arm (*P* = 0.03). The mechanism by which isoflavones can reduce PSA remains unclear. However, emerging evidence from molecular and histopathological studies posit that isoflavones may act as an anti-inflammatory agent contributing to a reduction in serum PSA [79–81]. However, the etiology and contribution of chronic inflammation in prostate carcinogenesis continues to remain unclear [79–81]. However, these differences were not observed among AAM, which may be attributed to the small sample size of AAM in this study. On the other hand, based on the significantly higher serum PSA concentrations observed in this population, we can attribute the lack of effect to the single dose and duration of intervention of the clinical trial.

LUTS represents a common conglomeration of storage, voiding, and post-micturition symptoms with potentially debilitating effects on quality of life [82–84]. Studies have demonstrated an increased prevalence of LUTS in men over age 60 and in those with benign prostatic hyperplasia (BPH) [82–84]. No significant differences between the treatment and placebo arms were observed in LUTS scores from baseline to end of study (data not shown), potentially attributed to absence of BPH in this subject population.

Our clinical trial had several strengths. The study was a randomized, placebo-controlled, double-blinded trial utilizing a standardized formulation of aglycone isoflavones and guided by an FDA IND, with comprehensive monitoring of safety, stringent criteria for eligibility and the rigor with which most therapeutic agents are developed. The study was in addition informed by population laboratory, animal studies and early phase trials demonstrating a rationale for further evaluating isoflavones for PCa chemoprevention. While these factors contributed to the rigor of the study design and conduct, the study is not without limitations. Accrual of AAM in the study was far lower than expected, and our study was ultimately underpowered to detect small reductions in specific biomarkers of disease progression in men with localized PCa. Future studies should ideally enroll larger cohorts of men from multi-institutional sites with experienced investigators and a larger pool of subjects to recruit from. The study, in addition used a “window of opportunity study design” providing an opportunity to evaluate agents for safety and efficacy signals using intermediate endpoint biomarkers of prostate cancer progression. The intervention window ranged from 3-6 weeks based on date of biopsy to date of prostatectomy, with an average number of subjects receiving agent for a minimum of 31 days. The duration of intervention was thus an added limitation to the underpowered study. Additionally, we did not observe a robust increase in plasma genistein. Our study, in addition did not include quantifying the composition of the gut microbiota and the potential contribution to absorption of genistein. Future studies should account for variability in absorption of soy foods in human clinical trials that have attributed this to intake of other nutrients that alter the gut flora, seasonal changes, timing of plasma and urinary isoflavone measurements due to the short half-life of these compounds, the composition of ethnic diets, and individual differences. Future studies should continue to examine both urinary metabolites as well as plasma levels of isoflavones.

## MATERIALS AND METHODS

The study and the consent procedures were approved by the institutional review boards of all participating institutions. A consort diagram depicting the number of subjects screened, enrolled, randomized and completed intervention is shown in Figure 1. Men between ages 30–80 with a biopsy-proven diagnosis of localized PCa (Gleason Score 6 (3+3)) before randomization, with no history of cancer, hepatic or renal disease, restricted from taking steroid or other supplements or more than 4 oz. of any isoflavones containing foods were eligible. All prostate biopsies were reviewed by a central pathology laboratory and all pathologists were unaware of the treatment-group assignment. Discordant interpretations were arbitrated by a referee pathologist (senior pathologist at Moffitt Cancer Center), and concordance was achieved in all cases. Participants were enrolled at the Moffitt Cancer Center, James A. Haley VA Hospital, Tampa and University of Florida, Jacksonville, Florida. Potential participants were identified by the primary surgeon and invited for eligibility screening. Screened subjects were recruited to the study on the day the surgeon informed the patient of the diagnosis and when patients opted for prostatectomy. Only subjects who had between 3–6 weeks (+/-3 days) from the date of randomization to prostatectomy were recruited in the trial. Additionally, confirmation of diagnosis by central pathology review and confirmation of inclusion and exclusion criteria and normal lab results were required for randomization. After eligibility was confirmed and consent obtained, CM and AAM were assigned to the intervention or placebo arm (1:1) using the SRAR system, a web-delivered subject registration application, stratified by race (AAM or CM). All study staff and participants, with the exception of the clinical pharmacist and biostatistician, were blinded to the assignments until the completion of the trial. Novasoy 400^®^, an investigational agent manufactured by Archer Daniels Midland Company, (Decatur, IL) was used in this clinical trial. Novasoy 400^®^ is a soy-based isoflavone concentrate extracted to assure that the ratio of isoflavones as well as the aglycone and glycoside isoforms is maintained as they would be found in soybeans and unfermented soy foods. The purified isoflavones provided by Archer Daniels Midland Company are standardized based on isoflavone content, and were maintained on a stability monitoring program to ensure that there is no reduction in active component during the period of use. Novasoy 400^®^ is combined with the filler Avicel PH105, a methyl cellulose blend and formulated to deliver 20 mg aglycone equivalent isoflavones per capsule. The placebo contains only Avicel PH105. Both are compounded in an opaque, gelatin capsule to conceal any differences between the active and placebo capsules. All active agent and placebo were in compliance with current good manufacturing practice regulations. An investigator-initiated IND (61,949 Kumar NB PI) was obtained for this agent at this dose and for this indication. Periodical testing was conducted to ensure drug stability with full potency of agent documented until end of trial. To minimize the use of other supplements, a standard vitamin and mineral formulation containing 100% U. S. recommended daily allowance was provided to all participants for the duration of the study. Change in percent Ki-67 expression, evaluated in PCa tissue specimens at baseline to 3–6 weeks (+/-3 days) of intervention with 20 mgs of aglycone isoflavones BID (40 mg daily) *vs*. placebo was the primary endpoint biomarker of efficacy. Immunohistochemistry (Ki-67) was performed on paraffin-embedded sections from prostate biopsies and prostatectomy tissues with prostate adenocarcinoma for evaluation of change in proliferation. Ki-67 antibody (clone MIB-1, DakoCytomation, Carpinteria, CA) was used at a dilution of 1:50. To enhance antigen retrieval, citrate buffer was used. The antigen antibody reaction was detected using DAB chromogen. The change in the percentage of Ki-67-positive tumor cells was evaluated. The primary safety endpoint is incidence and severity of AEs occurring during intervention with either purified isoflavones or placebo. All AEs that were reported by the subject, detected during a visit, physical examination, or laboratory work-up were recorded in the participant’s medical record and recorded on the CRF. All AEs that occurred once the subject is randomized and begins taking study agent were recorded on the AE CRF whether or not related to study agent. The following information were captured for each AE: date reported; verbatim term; CTCAE Term (v 5.0); onset and resolution date; severity grade; attribution to study agent; whether or not the event was reported as an SAE; action taken; whether or not the subject dropped due to the AE; outcome; and comments. Secondary endpoints included intermediate endpoint biomarkers of disease progression, serum steroid hormones, PSA, apoptotic index, Tumor Volume (TV), Gleason Score (GS); Symptoms including Lower Urinary Tract Symptoms (LUTS). At randomization, baseline assessments of lower urinary tract symptoms (LUTS) using the LUTS Symptoms Scale [82], PSA, serum steroid hormones (free and total testosterone, IGF-1, IGF-BP-3 and total estradiol), tumor volume, (TV), percent Ki-67 and Gleason Score (GS) and plasma isoflavone levels were obtained. LUTS [37], plasma isoflavone concentrations, serum steroid hormones, levels, PSA and nutritional intake data were evaluated at baseline, midpoint and at end-of-study (EOS). Monthly assessments of toxicity (CTCAE 5.0), concomitant medications and organ function, including CBC and CMP were performed. All participants in the study had EOS prostatectomy. Any toxicities (adverse events) occurring during the study were reviewed by the treating physician and managed according to standard medical practice. The intervention was terminated if a participant developed a serious adverse event. All subjects were contacted 7±3 days following the 6 week intervention to assess toxicity and concomitant medications. Compliance with study agent intake was measured during monthly visits via pill counts and self-reported daily study-agent intake logs. Adherence was assessed by measuring plasma isoflavone, specifically genistein levels at baseline and EOS. A validated liquid chromatography triple quadrupole mass spectrometry (LC/MS/MS) method (Thermo Scientific, San Jose, CA) was used to determine plasma isoflavone, genistein levels. Toxicities were monitored continuously through the trial by the PI and study physician at each site. The study was monitored in accordance with the Protocol Review and Monitoring System at the Moffitt Cancer Center and an External Data and Safety Monitoring Board (EDSMB).

### Statistical methods

The original proposal was to recruit 96 men, with 48 subjects in the treatment arm (12 AA and 36 CA) receiving isoflavones and 48 subjects receiving placebo (12 AA and 36 CA). We identified Ki-67, a proliferation index associated with disease progression as the primary endpoint in this trial. The percentage change in % Ki-67 expression from pre- to post-treatment in African-American men to Caucasian men were made using the Wilcoxon rank sum test. With alpha equal to .05 and 3 times as many Caucasians as African-Americans, we planned a sample size to provide 80 percent power with a sample size of 48 subjects in the treated arm, assuming an effect size of 1.

Of a total of 128 men meeting all eligibility requirements, 71 were randomized on study (Figure 1). Thirty six participants (25 CM, 6AAM) were randomized to the isoflavone arm and 34 (25 CM, 7AAM) to the placebo arm, with 62 completing the intervention. Since the accrual of AAM in the study was far lower than expected, our study was ultimately underpowered to detect small changes in specific biomarkers of disease progression proposed in men with localized PCa.

The primary data analysis focused on the changes in biomarkers for PCa progression from baseline to prostatectomy using tissue and serum samples. These endpoints were analyzed and compared. The primary comparisons were those between the treated vs. placebo arms within each racial group. In addition, we will compare the treatment effect between two racial groups as a secondary objective. That is, we evaluated whether there is any interaction between the treatment and race factor.

Baseline participant characteristics were compared between the two groups using Fisher exact tests for categorical variables and Wilcoxon rank-sum test for continuous variables. Adverse events by group, grade and causality were compared using the Wilcoxon rank-sum test. Plasma isoflavone levels, serum steroid hormone levels, nutritional intake, LUTS and QOL were compared by study arm from baseline to end of intervention using 2-sided Wilcoxon rank-sum test. We estimated the overall treatment effect on serum PSA, steroid hormones between the two arms using the Wilcoxon rank-sum test.

## CONCLUSIONS

In conclusion, a daily intake of a standardized isoflavone formulation (20 mgs of aglycones BID) for 3–6 weeks was well tolerated and accumulated in plasma. However, the formulation, dose and duration of intervention failed to reduce tissue markers of proliferation in men with localized PCa. A significant reduction in serum PSA was observed with isoflavone supplementation in CM compared to the placebo arm, although these reductions were not observed in AAM. We observed no changes in serum steroid hormones with isoflavone supplementation. There were some interesting findings with serum IGF-1 concentrations and IGF1: IGFBP-3 ratio in AAM that may be worth examining in a larger cohort of AAM using multiple doses and a longer duration of intervention. Prior to recommending isoflavones for prevention of PCa, clinicians must exercise caution until further evaluation of isoflavones in well-powered clinical trials is completed.
